# Modeling survival in colon cancer: a methodological review

**DOI:** 10.1186/1476-4598-6-15

**Published:** 2007-02-12

**Authors:** Farid E Ahmed, Paul W Vos, Don Holbert

**Affiliations:** 1 Department of Radiation Oncology, The Brody School of Medicine and East Carolina University, 600 Moye Blvd., LSB 014, Greenville, NC 27858 USA; 2 Department of Biostatistics, School of Allied Health Sciences, East Carolina University, Greenville, NC 27858 USA

## Abstract

The Cox proportional hazards model is the most widely used model for survival analysis because of its simplicity. The fundamental assumption in this model is the proportionality of the hazard function. When this condition is not met, other modifications or other models must be used for analysis of survival data. We illustrate in this review several methodological approaches to deal with the violation of the proportionality assumption, using survival in colon cancer as an illustrative example.

## Background

Several methods for estimating survival probability of populations from patient samples have been proposed since the first systematic approach in 1950 [1]. One of the oldest and most straightforward methods for analyzing survival data is to compute the Life Table. This method, proposed by Berkson and Gage [1] for studying cancer survival, uses an enhanced frequency distribution approach. To compute a Life Table, the range of survival times for all patients is divided into subintervals. For each interval, one computes the number and proportion of cases that entered the interval "alive." the number and proportion of cases that "died", and the number of cases that were lost or "censored" in the respective time interval. An observation is censored if the subject leaves the study or is alive when the study ends. Appropriate manipulation of these quantities allows estimation of parameters of interest related to the survival distribution.

**Figure 2:**
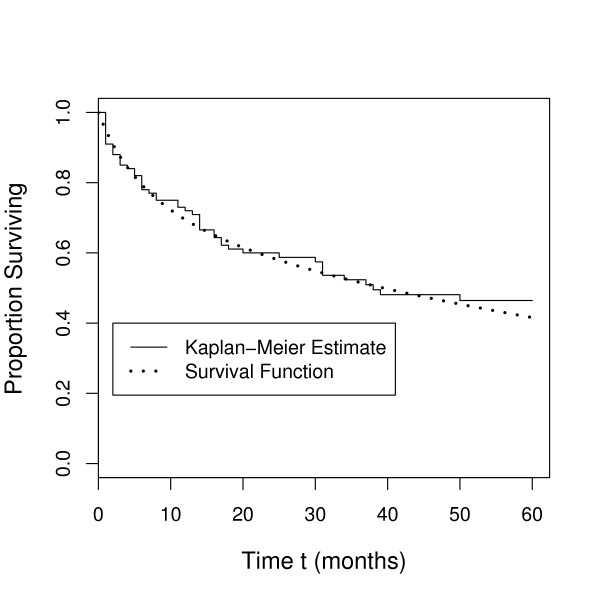
**Survival Function and Kaplan-Meier Estimate**. Kaplan-Meier estimate (step curve) of the survival function and the population survival function (dotted curve). The survival function for the population is known because these data were simulated.

While the Life Table method worked well for a homogeneous sample, it did not address a primary goal of cancer research, namely to determine whether or not certain continuous and/or categorical variables are related to the survival times. This need led to the application of regression methods for analyzing survival data. The standard multiple linear regression model is not well suited to survival data for several reasons; among these are (i) survival times are typically not normally distributed, and (ii) censored data is commonplace, resulting in missing values for the dependent variable (survival time). Early attempts to circumvent these problems involved applying the log transform to survival time, but this worked well only when censoring was present in a very small percentage of the observations (Everitt and Rabe-Hesketh, [2]). Two important developments that have greatly enhanced survival analysis methods are the derivation of a nonparametric method for constructing a survival curve from censored data by Kaplan and Meier [3], and the Proportional Hazards (PH) model proposed by Cox [4]. With the rapid improvements in the graphics capabilities of personal computers over the last 20 years, the use of the Kaplan-Meier method has become so popular that survival curves are often referred to as "Kaplan-Meier curves". An example of a survival curve estimated using the Kaplan-Meier approach is shown in Figure 2. The Cox model, a rnultivariate semiparametric regression model, is now the most widely used in clinical studies to characterize disease progression on existing cases by revealing the importance of covariates. It is the most popular model for survival analysis due to its simplicity. The proportional hazards model is the most general of the regression models because it is not based on any assumptions concerning the nature or shape of the underlying-survival distribution. The model assumes that the underlying hazard rate (rather than survival time) is a function of the independent variables (covariates); no assumptions are made about the nature or shape of the hazard function.

The hazard function (developed more fully in section 2) is a non-negative function (of time) which can be thought of as reflecting the change in an individual's probability of death in the immediate future, given that the individual has survived up to the current time. In humans, the probability of death is higher immediately after birth than it is when the newborn becomes more mature; consequently, the hazard function decreases with age through the maturation process. Subsequent to maturation, there is a long period where an individual's probability of death (in the immediate future) is relatively low and changing very little with the passage of time; here, the hazard function is rather flat and closer to zero. In the final stages of life, this probability increases with increasing age, resulting in an increase in the hazard function.

This pattern of change gives rise to a U-shaped (or "bathtub shaped") hazard function for all cause mortality in humans. The hazard is sometimes referred to as the "force of mortality" or the "conditional failure rate".

Mathematically, the hazard function is simply a re-expression of the survival function, in that specification of either one of these uniquely determines the other. The hazard function, however, has more visual appeal in that it directly displays the time periods over which changes to the risk of death in the immediate future are occurring. To identify these periods from the survival function (rather than the hazard function), the analyst would have to look for sharp drops and flatter sections of the survival curve. The hazard function displays the information more directly.

The Cox model relates the hazard function of an individual at time *t*, with a vector *X *= (*X*_1_, *X*_2_,..., *X*_*p*_) of *p *covariates (explanatory or predictor variables), to a baseline hazard function *h*_0_(*t*) via a log-linear function:

h(t;X)=h0(t)exp⁡{∑j=1pβjXj},     (1)
 MathType@MTEF@5@5@+=feaafiart1ev1aaatCvAUfKttLearuWrP9MDH5MBPbIqV92AaeXatLxBI9gBaebbnrfifHhDYfgasaacH8akY=wiFfYdH8Gipec8Eeeu0xXdbba9frFj0=OqFfea0dXdd9vqai=hGuQ8kuc9pgc9s8qqaq=dirpe0xb9q8qiLsFr0=vr0=vr0dc8meaabaqaciaacaGaaeqabaqabeGadaaakeaacqWGObaAcqGGOaakcqWG0baDcqGG7aWocqWGybawcqGGPaqkcqGH9aqpcqWGObaAdaWgaaWcbaGaeGimaadabeaakiabcIcaOiabdsha0jabcMcaPiGbcwgaLjabcIha4jabcchaWnaacmqabaWaaabCaeaaiiGacqWFYoGydaWgaaWcbaGaemOAaOgabeaakiabdIfaynaaBaaaleaacqWGQbGAaeqaaaqaaiabdQgaQjabg2da9iabigdaXaqaaiabdchaWbqdcqGHris5aaGccaGL7bGaayzFaaGaeiilaWIaaCzcaiaaxMaadaqadaqaaiabigdaXaGaayjkaiaawMcaaaaa@5202@

where *β *= (*β*_1_, *β*_2_,..., *β*_*p*_) is a vector of coefficients. An important consequence of this formulation, and the reason for the name "Proportional Hazards Model", is that the hazard ratio function (HRF) for two individuals with covariates *X *and X* does not depend on time; *h*(*t*; *X*) is a constant multiple of *h*(*t*; X*). It is also important to note that the effects of the covariates on the hazard are assumed to be constant over time. Inference about the regression parameter (*β*) is possible without making assumptions about the form of the baseline hazard function, *h*_0_(*t*); the hypothesis of no association of one or more of the *p *independent variables with survival can be tested by the likelihood ratio test (LRT) [4].

**Figure 1:**
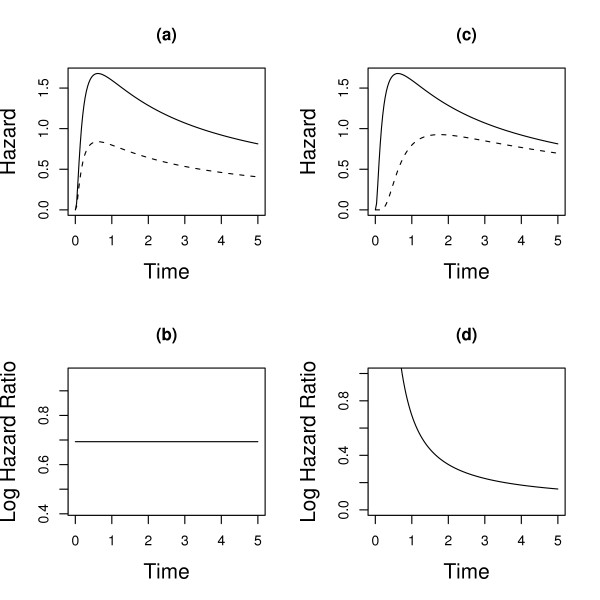
**Hazard Functions**. Two hazard functions that are proportional are shown in (a); the log of their ratio is constant in time as seen in (b). Two nonproportional hazard functions are shown in (c); the log of their ratio depends on time as seen in (d).

Figure 1 illustrates the difference between proportional hazards and non-proportional hazards. In Figure 1a the two hazard functions are proportional, and their corresponding HRF (and log hazard ratio function, LHRF) is constant over time as shown in Figure 1b. On the other hand, if the hazard functions are not proportional, they might start at the same value and then diverge, or converge to some common value (Figure 1c), or cross and diverge again, and the corresponding LHRF will not be constant (Figure 1d). Many possibilities arise due to this non-proportionality. For example, the resulting LHRFs may be linear, non linear but monotonic (e.g. logarithmic), or non-monotonic (e.g. quadratic) (Ohno-Machado [5]).

In most applications of the Cox model in cancer research, the goal is to compare survival characteristics between two or more treatment groups. This is accomplished by letting one (or more) of the covariates serve as group indicator variable(s). For example, in comparing a treated group with a control group, we might assign *X*_1 _= 0 for control group cases and *X*_1 _= 1 for treated group cases, and compare the fitted survival curves. In this setting, it follows from the constancy of the HRF that the survival curves for different groups do not cross. In practice, however, the HRF may change over time leading to incorrect inferences (O' Quigley and Pessione [6] and Hess [7]) if the Cox model were applied. Although there are several tests available to check for PH violations (Lin *et al *[8]), they are rarely used because after rejecting the assumption it may not be known how to model the effect of the predictors (Quantin *et al *[9]).

This article focuses on a review of (a) the Cox model and interpretation of its parameters, (b) assessment of the validity of the PH assumption, (c) the use of time-varying coefficients, and (d) accommodating nonproportional hazards using covariate stratification, partitioning of the time axis, and modeling tune dependence of the regression coefficients.

## 1 Proportional Hazards Model

We describe the models in this paper using a synthetic colon cancer survival (SCCS) data set on 100 individuals. The explanatory variables considered are Treatment taking values 1 or 2, Race taking values W and B, and Stage taking values 1,2, and 3. The survival time in months is recorded (Time) and whether the observation was censored (Event = 0). Data for the first five individuals appear in Table 1. For example, subject 1 in this table died after 3 months of followup whereas subject 3 was still alive after 60 months of followup. The number of censored events is 51 and the median of the variable Time is 24. The estimated median survival time will be higher than the sample median because of the censored observations. The explanatory variables are summarized as Treatment: 47 received 'Treatment 1', 53 received 'Treatment 2', Race: 29 were 'Black', 71 were 'White', and Stage: 44 were 'Stage 1', 29 were 'Stage 2', and 27 were 'Stage 3'.

**Table 1:** First five observations from SCCS

	Time	Event	Treatment	Race	Stage
1	3	1	1	W	1
2	8	1	1	W	3
3	60	0	2	W	1
4	48	0	2	W	3
5	2	1	2	B	3

The proportional hazards model as well as the models for nonproportional hazards attempt to describe the survival function *S*(*t*) which is the proportion of individuals in the population surviving beyond time *t*,

S(t)=number of individuals surviving longer than time ttotal number of individuals in the population.
 MathType@MTEF@5@5@+=feaafiart1ev1aaatCvAUfKttLearuWrP9MDH5MBPbIqV92AaeXatLxBI9gBaebbnrfifHhDYfgasaacH8akY=wiFfYdH8Gipec8Eeeu0xXdbba9frFj0=OqFfea0dXdd9vqai=hGuQ8kuc9pgc9s8qqaq=dirpe0xb9q8qiLsFr0=vr0=vr0dc8meaabaqaciaacaGaaeqabaqabeGadaaakeaacqWGtbWucqGGOaakcqWG0baDcqGGPaqkcqGH9aqpdaWcaaqaaiabb6gaUjabbwha1jabb2gaTjabbkgaIjabbwgaLjabbkhaYjabbccaGiabb+gaVjabbAgaMjabbccaGiabbMgaPjabb6gaUjabbsgaKjabbMgaPjabbAha2jabbMgaPjabbsgaKjabbwha1jabbggaHjabbYgaSjabbohaZjabbccaGiabbohaZjabbwha1jabbkhaYjabbAha2jabbMgaPjabbAha2jabbMgaPjabb6gaUjabbEgaNjabbccaGiabbYgaSjabb+gaVjabb6gaUjabbEgaNjabbwgaLjabbkhaYjabbccaGiabbsha0jabbIgaOjabbggaHjabb6gaUjabbccaGiabbsha0jabbMgaPjabb2gaTjabbwgaLjabbccaGiabdsha0bqaaiabbsha0jabb+gaVjabbsha0jabbggaHjabbYgaSjabbccaGiabb6gaUjabbwha1jabb2gaTjabbkgaIjabbwgaLjabbkhaYjabbccaGiabb+gaVjabbAgaMjabbccaGiabbMgaPjabb6gaUjabbsgaKjabbMgaPjabbAha2jabbMgaPjabbsgaKjabbwha1jabbggaHjabbYgaSjabbohaZjabbccaGiabbMgaPjabb6gaUjabbccaGiabbsha0jabbIgaOjabbwgaLjabbccaGiabbchaWjabb+gaVjabbchaWjabbwha1jabbYgaSjabbggaHjabbsha0jabbMgaPjabb+gaVjabb6gaUbaacqGGUaGlaaa@ADCD@

For an individual selected at random from the population, the survival function can be interpreted as *the probability this individual survives beyond time t*.

For real data the survival function is not known but can be estimated from a sample. If everyone in the sample were observed until death the survival function could be estimated by

S^(t)=number of individuals surviving longer than time ttotal number of individuals in the sample
 MathType@MTEF@5@5@+=feaafiart1ev1aaatCvAUfKttLearuWrP9MDH5MBPbIqV92AaeXatLxBI9gBaebbnrfifHhDYfgasaacH8akY=wiFfYdH8Gipec8Eeeu0xXdbba9frFj0=OqFfea0dXdd9vqai=hGuQ8kuc9pgc9s8qqaq=dirpe0xb9q8qiLsFr0=vr0=vr0dc8meaabaqaciaacaGaaeqabaqabeGadaaakeaacuWGtbWugaqcaiabcIcaOiabdsha0jabcMcaPiabg2da9maalaaabaGaeeOBa4MaeeyDauNaeeyBa0MaeeOyaiMaeeyzauMaeeOCaiNaeeiiaaIaee4Ba8MaeeOzayMaeeiiaaIaeeyAaKMaeeOBa4MaeeizaqMaeeyAaKMaeeODayNaeeyAaKMaeeizaqMaeeyDauNaeeyyaeMaeeiBaWMaee4CamNaeeiiaaIaee4CamNaeeyDauNaeeOCaiNaeeODayNaeeyAaKMaeeODayNaeeyAaKMaeeOBa4Maee4zaCMaeeiiaaIaeeiBaWMaee4Ba8MaeeOBa4Maee4zaCMaeeyzauMaeeOCaiNaeeiiaaIaeeiDaqNaeeiAaGMaeeyyaeMaeeOBa4MaeeiiaaIaeeiDaqNaeeyAaKMaeeyBa0MaeeyzauMaeeiiaaIaemiDaqhabaGaeeiDaqNaee4Ba8MaeeiDaqNaeeyyaeMaeeiBaWMaeeiiaaIaeeOBa4MaeeyDauNaeeyBa0MaeeOyaiMaeeyzauMaeeOCaiNaeeiiaaIaee4Ba8MaeeOzayMaeeiiaaIaeeyAaKMaeeOBa4MaeeizaqMaeeyAaKMaeeODayNaeeyAaKMaeeizaqMaeeyDauNaeeyyaeMaeeiBaWMaee4CamNaeeiiaaIaeeyAaKMaeeOBa4MaeeiiaaIaeeiDaqNaeeiAaGMaeeyzauMaeeiiaaIaee4CamNaeeyyaeMaeeyBa0MaeeiCaaNaeeiBaWMaeeyzaugaaaaa@A74B@

Typically, not all individuals are observed until death so that some of the data are censored. One method for dealing with censored data is to estimate the survival function with the Kaplan-Meier estimator. The Kaplan-Meier estimate for the data in SCCS is plotted in Figure 2. This estimate can be described as a decreasing step function where the steps occur when a death has been observed. The smooth function is the true survival function which we know in this case because the data have been simulated. This population survival function is for a population with the following distributions: Treatment (50% Treatment 1, 50% Treatment 2), Race (80% White, 20% Black), and Stage (40% Stage 1, 30% Stage 2, 30% Stage3).

The survival function shows the relationship between survival and time; that is, between the variables Time and Event in the SCCS data set. To describe the effect of other variables, such as Treatment. Race, and Stage, on survival it is convenient to use the hazard function.

The hazard function addresses the question:

What is the probability that an individual who has survived to time *t *dies in the interval *t *to *t *+ Δ*t*?

As the interval Δ*t *gets close to zero so does the probability of death and the hazard function *h*(*t*) is the rate at which the probability goes to zero:

h(t)=lim⁡Δt→0probability of an individual alive at time t dies between tand t+ΔtΔt.
 MathType@MTEF@5@5@+=feaafiart1ev1aaatCvAUfKttLearuWrP9MDH5MBPbIqV92AaeXatLxBI9gBaebbnrfifHhDYfgasaacH8akY=wiFfYdH8Gipec8Eeeu0xXdbba9frFj0=OqFfea0dXdd9vqai=hGuQ8kuc9pgc9s8qqaq=dirpe0xb9q8qiLsFr0=vr0=vr0dc8meaabaqaciaacaGaaeqabaqabeGadaaakeaacqWGObaAcqGGOaakcqWG0baDcqGGPaqkcqGH9aqpdaWfqaqaaiGbcYgaSjabcMgaPjabc2gaTbWcbaGaeuiLdqKaemiDaqNaeyOKH4QaeGimaadabeaakmaalaaabaGaeeiCaaNaeeOCaiNaee4Ba8MaeeOyaiMaeeyyaeMaeeOyaiMaeeyAaKMaeeiBaWMaeeyAaKMaeeiDaqNaeeyEaKNaeeiiaaIaee4Ba8MaeeOzayMaeeiiaaIaeeyyaeMaeeOBa4MaeeiiaaIaeeyAaKMaeeOBa4MaeeizaqMaeeyAaKMaeeODayNaeeyAaKMaeeizaqMaeeyDauNaeeyyaeMaeeiBaWMaeeiiaaIaeeyyaeMaeeiBaWMaeeyAaKMaeeODayNaeeyzauMaeeiiaaIaeeyyaeMaeeiDaqNaeeiiaaIaeeiDaqNaeeyAaKMaeeyBa0MaeeyzauMaeeiiaaIaemiDaqNaeeiiaaIaeeizaqMaeeyAaKMaeeyzauMaee4CamNaeeiiaaIaeeOyaiMaeeyzauMaeeiDaqNaee4DaCNaeeyzauMaeeyzauMaeeOBa4MaeeiiaaccbiGae8hDaqNae8hiaaIaeeyyaeMaeeOBa4MaeeizaqMaeeiiaaIaemiDaqNaey4kaSIaeuiLdqKaemiDaqhabaGaeuiLdqKaemiDaqhaaiabc6caUaaa@9559@

The hazard function *h*(*t*) is not a probability so it can be larger than one and its value will depend on the units used for time (e.g., days or months). The hazard function is used to give an approximate probability, namely *h*(*t*)Δ*t*, for the event that an individual dies in interval *t *to *t *+ Δ*t*. This approximation is very good for small Δ*t*. The hazard function is also related to the survival function. In fact, the hazard function is an equivalent formulation for the relationship between survival time and the event of death. The proportional hazards model is a simple model to describe the survival between two groups having hazard functions *h*_1_(*t*) and *h*_2_(*t*). The proportional hazards assumption is that ratio of these hazard functions does not depend on time:

ψ=h2(t)h1(t)
 MathType@MTEF@5@5@+=feaafiart1ev1aaatCvAUfKttLearuWrP9MDH5MBPbIqV92AaeXatLxBI9gBaebbnrfifHhDYfgasaacH8akY=wiFfYdH8Gipec8Eeeu0xXdbba9frFj0=OqFfea0dXdd9vqai=hGuQ8kuc9pgc9s8qqaq=dirpe0xb9q8qiLsFr0=vr0=vr0dc8meaabaqaciaacaGaaeqabaqabeGadaaakeaaiiGacqWFipqEcqGH9aqpdaWcaaqaaiabdIgaOnaaBaaaleaacqaIYaGmaeqaaOGaeiikaGIaemiDaqNaeiykaKcabaGaemiAaG2aaSbaaSqaaiabigdaXaqabaGccqGGOaakcqWG0baDcqGGPaqkaaaaaa@3ADD@

where *ψ *is called the proportionality constant. If *ψ *> 1 Individuals in group 2 have a greater risk of dying than individuals in group 1, regardless of the time *t*.

While *ψ *does not depend on time, it does depend on the explanatory variables in the model, namely Treatment, Race, and Stage. A convenient and readily interpretable linear expression for the logarithm of *ψ *(which can be any real number, since *ψ *> 0) is obtained by the proper use of indicator variables according to the following scheme:

For Treatment:

xTrt2={0if Treatment is 11if Treatment is 2
 MathType@MTEF@5@5@+=feaafiart1ev1aaatCvAUfKttLearuWrP9MDH5MBPbIqV92AaeXatLxBI9gBaebbnrfifHhDYfgasaacH8akY=wiFfYdH8Gipec8Eeeu0xXdbba9frFj0=OqFfea0dXdd9vqai=hGuQ8kuc9pgc9s8qqaq=dirpe0xb9q8qiLsFr0=vr0=vr0dc8meaabaqaciaacaGaaeqabaqabeGadaaakeaacqWG4baEdaWgaaWcbaGaeeivaqLaeeOCaiNaeeiDaqNaeGOmaidabeaakiabg2da9maaceqabaqbaeaabiGaaaqaaiabicdaWaqaaiabbMgaPjabbAgaMjabbccaGiabbsfaujabbkhaYjabbwgaLjabbggaHjabbsha0jabb2gaTjabbwgaLjabb6gaUjabbsha0jabbccaGiabbMgaPjabbohaZjabbccaGiabigdaXaqaaiabigdaXaqaaiabbMgaPjabbAgaMjabbccaGiabbsfaujabbkhaYjabbwgaLjabbggaHjabbsha0jabb2gaTjabbwgaLjabb6gaUjabbsha0jabbccaGiabbMgaPjabbohaZjabbccaGiabikdaYaaaaiaawUhaaaaa@6122@

For Race:

xRaceW={0if Race is B1if Race is W
 MathType@MTEF@5@5@+=feaafiart1ev1aaatCvAUfKttLearuWrP9MDH5MBPbIqV92AaeXatLxBI9gBaebbnrfifHhDYfgasaacH8akY=wiFfYdH8Gipec8Eeeu0xXdbba9frFj0=OqFfea0dXdd9vqai=hGuQ8kuc9pgc9s8qqaq=dirpe0xb9q8qiLsFr0=vr0=vr0dc8meaabaqaciaacaGaaeqabaqabeGadaaakeaacqWG4baEdaWgaaWcbaGaeeOuaiLaeeyyaeMaee4yamMaeeyzauMaee4vaCfabeaakiabg2da9maaceqabaqbaeaabiGaaaqaaiabicdaWaqaaiabbMgaPjabbAgaMjabbccaGiabbkfasjabbggaHjabbogaJjabbwgaLjabbccaGiabbMgaPjabbohaZjabbccaGiabbkeacbqaaiabigdaXaqaaiabbMgaPjabbAgaMjabbccaGiabbkfasjabbggaHjabbogaJjabbwgaLjabbccaGiabbMgaPjabbohaZjabbccaGiabbEfaxbaaaiaawUhaaaaa@54A2@

For Stage:

*x*_Stg2 _= 0 and *x*_Stg3 _= 0   if Stage is 1

*x*_Stg2 _= 1 and *x*_Stg3 _= 0   if Stage is 2

*x*_Stg2 _= 0 and *x*_Stg3 _= 1   if Stage is 3

With this coding of the explanatory variables, and appropriate substitution into equation 1, it can be shown that

log(*ψ*) = *β*_Trt2_*x*_Trt2 _+ *β*_RaceW_*x*_RaceW _+ *β*_Stg2_*x*_Stg2 _+ *β*_Stg3_*x*_Stg3 _    (2)

where the *β*'s have been subscripted to reflect their role in the hypothesized causal mechanism, and the subscript on *x *indicates when the variable takes the value "1" rather than "0". For example. *x*_Trt2_, *x*_RaceW_, and *x*_Stg2 _are all 1 and *x*_Stg3 _is 0 when the individual receives treatment 2, is white and has stage 2 cancer. An individual who received treatment 1, was black, and had stage 1 cancer would have each of these explanatory variables equal to zero. If *β*_Trt2 _> 0, individuals receiving treatment 2 have a larger log hazard function than those receiving treatment 1. Expressed in terms of the hazard function, exp(*β*_Trt2_) > 1 indicates that the hazard function for treatment 2 is proportionally greater than that of treatment 1 and the proportionality is constant for all time *t*. The generic expression of the hazards proportionality constant is

log⁡(ψ)=β1x1+β2x2+⋯+βpxp=∑i=1pβixi.
 MathType@MTEF@5@5@+=feaafiart1ev1aaatCvAUfKttLearuWrP9MDH5MBPbIqV92AaeXatLxBI9gBaebbnrfifHhDYfgasaacH8akY=wiFfYdH8Gipec8Eeeu0xXdbba9frFj0=OqFfea0dXdd9vqai=hGuQ8kuc9pgc9s8qqaq=dirpe0xb9q8qiLsFr0=vr0=vr0dc8meaabaqaciaacaGaaeqabaqabeGadaaakeaacyGGSbaBcqGGVbWBcqGGNbWzcqGGOaakiiGacqWFipqEcqGGPaqkcqGH9aqpcqWFYoGydaWgaaWcbaGaeGymaedabeaakiabdIha4naaBaaaleaacqaIXaqmaeqaaOGaey4kaSIae8NSdi2aaSbaaSqaaiabikdaYaqabaGccqWG4baEdaWgaaWcbaGaeGOmaidabeaakiabgUcaRiabl+UimjabgUcaRiab=j7aInaaBaaaleaacqWGWbaCaeqaaOGaemiEaG3aaSbaaSqaaiabdchaWbqabaGccqGH9aqpdaaeWbqaaiab=j7aInaaBaaaleaacqWGPbqAaeqaaOGaemiEaG3aaSbaaSqaaiabdMgaPbqabaaabaGaemyAaKMaeyypa0JaeGymaedabaGaemiCaahaniabggHiLdGccqGGUaGlaaa@5A16@

Fitting the proportional hazards model in (2) gives the estimates in Table 2. This model indicates that the effect of receiving treatment 2 rather than 1 is to decrease the hazard rate. That is, if we compare two groups one receiving treatment 1 and the other treatment 2, but the groups have the same values for the other explanatory variables, then the hazard rate for the group receiving treatment 2 is 0.46 times the hazard rate for those receiving treatment 1. The effect of being white is to decrease the hazard rate by the factor 0.4. Similar interpretation can be given for the effects of Stage2 and Stage3.

**Table 2:** Parameter estimates for the proportional hazards model using SCCS data

	coef	exp(coef)	se(coef)	z	P
Treatment2	-0.78	0.46	0.31	-2.53	0.01
RaceW	-0.92	0.40	0.30	-3.09	0.00
Stage2	-1.05	0.35	0.39	-2.69	0.01
Stage3	0.05	1.05	0.34	0.15	0.88

Since the validity of inferences based on the Cox model depends on the proportional hazards assumption, it is desirable to have diagnostic methods for checking this assumption. Many tools are available for checking the PH assumption. These include:

### (i) Plots of log(-log *S*(*t*))

Suppose the hazards for 2 groups are proportional, say *h*_1_(*t*) = *ψh*_2_(*t*). It can be shown that this results in the relationship log(-log *S*_1_(*t*)) = log *ψ *+ log(-log *S*_2_(*t*)); the log(-log *S*(*t*)) curves for the two groups differ by a constant. Plotting the estimated log(-log *S*(*t*)) curves for the two groups, evaluated at the covariate mean values, provides a visual check of the PH assumption. A clear departure from parallelism of these two curves would be consistent with violation of the PH assumption.

### (ii) Interactions with Time

Interaction of a covariate *x *with time can be modeled by including in the model a product term *x *× (time), resulting in a log hazard function of the form

log *h*(*t*) = ⋯ + *β*_1_*x *+ *β*_2_*xt *+ ⋯ = ⋯ + (*β*_1 _+ *β*_2_*t*)*x *+ ⋯

The coefficient *β*_2 _reflects *x*'s dependence on time; if this effect is statistically significant in the fitted model we have evidence for non-proportionality.

### (iii) Schoenfeld Partial Residuals

For each covariate, a Schoenfeld residual can be calculated for each case that was not censored. A plot of these residuals against time should be "approximately flat" if the PH assumption holds. We illustrate a check on the proportionality assumption for the SCSS dataset using a Schoenfeld residual plot with a smooth curve fit to these residuals (Grambsch and Therneau [10]).

**Figure 3:**
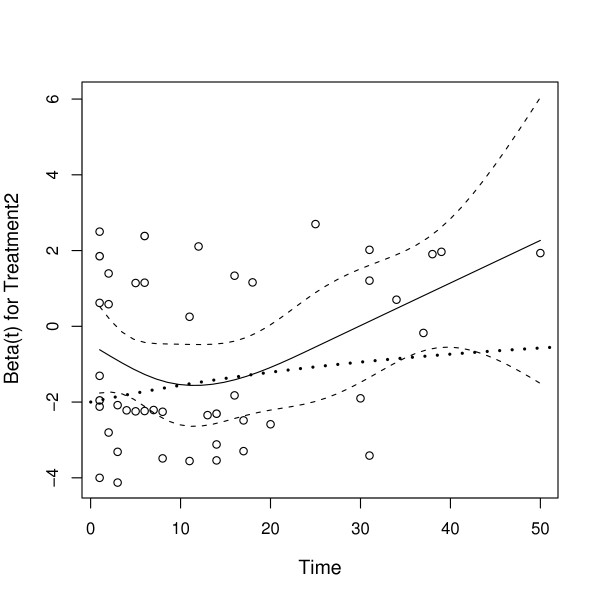
**Schoenfeld Residual Plot for *β*_Trt2_**. Schoenfeld residual plot for *β*_Trt2_. The black solid line is β^
 MathType@MTEF@5@5@+=feaafiart1ev1aaatCvAUfKttLearuWrP9MDH5MBPbIqV92AaeXatLxBI9gBaebbnrfifHhDYfgasaacH8akY=wiFfYdH8Gipec8Eeeu0xXdbba9frFj0=OqFfea0dXdd9vqai=hGuQ8kuc9pgc9s8qqaq=dirpe0xb9q8qiLsFr0=vr0=vr0dc8meaabaqaciaacaGaaeqabaqabeGadaaakeaaiiGacuWFYoGygaqcaaaa@2E64@_Trt2_, a smooth function fit to the residuals, and the dotted line shows the actual dependence of *β*_Trt2 _on *t *(which is known in this case because the data have been simulated).

**Figure 4:**
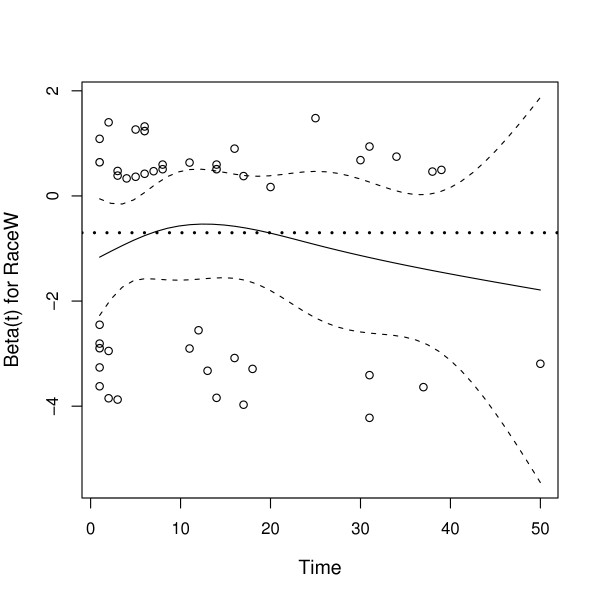
**Schoenfeld residual plot for *β*_RaceW_**. Schoenfeld residual plot for *β*_RaceW_. The black solid line is β^
 MathType@MTEF@5@5@+=feaafiart1ev1aaatCvAUfKttLearuWrP9MDH5MBPbIqV92AaeXatLxBI9gBaebbnrfifHhDYfgasaacH8akY=wiFfYdH8Gipec8Eeeu0xXdbba9frFj0=OqFfea0dXdd9vqai=hGuQ8kuc9pgc9s8qqaq=dirpe0xb9q8qiLsFr0=vr0=vr0dc8meaabaqaciaacaGaaeqabaqabeGadaaakeaaiiGacuWFYoGygaqcaaaa@2E64@_RaceW_, a smooth function fit to the residuals; *β*_RaceW _is constant in time which is indicated by the horizontal dotted line (which is known in this case because the data have been simulated).

Figure 3 is the Schoenfeld residual plot for *β*_Trt2_. The dashed lines are confidence bands drawn at plus and minus two standard errors. The dotted line shows the true functional dependence of *β*_Trt2 _on time *t*. Note that *β*_Trt2_(*t*) is not constant but decreases toward zero as time increases. That is, the treatment effect dies off over time. The estimated curve indicates that β^
 MathType@MTEF@5@5@+=feaafiart1ev1aaatCvAUfKttLearuWrP9MDH5MBPbIqV92AaeXatLxBI9gBaebbnrfifHhDYfgasaacH8akY=wiFfYdH8Gipec8Eeeu0xXdbba9frFj0=OqFfea0dXdd9vqai=hGuQ8kuc9pgc9s8qqaq=dirpe0xb9q8qiLsFr0=vr0=vr0dc8meaabaqaciaacaGaaeqabaqabeGadaaakeaaiiGacuWFYoGygaqcaaaa@2E64@_Trt2_(*t*) changes sign for large time *t*. The true parameter *β*_RaceW_(*t*) is constant in time but the estimate β^
 MathType@MTEF@5@5@+=feaafiart1ev1aaatCvAUfKttLearuWrP9MDH5MBPbIqV92AaeXatLxBI9gBaebbnrfifHhDYfgasaacH8akY=wiFfYdH8Gipec8Eeeu0xXdbba9frFj0=OqFfea0dXdd9vqai=hGuQ8kuc9pgc9s8qqaq=dirpe0xb9q8qiLsFr0=vr0=vr0dc8meaabaqaciaacaGaaeqabaqabeGadaaakeaaiiGacuWFYoGygaqcaaaa@2E64@_RaceW_(*t*) indicates this parameter could depend on *t*. See Figure 4.

## 2 Dealing with Nonproportional Hazards

In the SCCS example from the previous section we encountered a model with a nonpreportional hazard function where one of the coefficients varied with time *t*. In this section we address some ways to cope with time varying coefficients *β*(*t*) in the Cox model. Before describing these methods we list the following considerations and caveats.

• Detecting the true time dependence of the parameter can be difficult. The Schoenfeld plot for Treatment in Figure 3 shows that the parameter is not constant but does not indicate the proper functional form. Therneau and Grambsch [11] give examples where the data cannot distinguish two very different functions of time.

• While there are tests to check for nonproportionality (see for example [10]), these need not be effective against some alternatives. In particular, tests based on the slope of *β*(*t*) need not be sensitive to quadratic or other nonlinear functions of time.

• Even when a test for nonproportionality is statistically significant, this does not mean the nonproportionality is of practical importance or significance. This potential difficulty arises in any significance test, especially if the sample is large.

• Time varying coefficients can be due to other inadequacies in the model. For example, one or more covariates are not included, the functional form of the covariates is incorrect, or another survival function can more effectively model the data.

We consider three methods for dealing with time-varying coefficients: stratification of variables with time varying coefficients, partitioning of the time axis, and modeling the dependence of *β*(*t*) on time. We discuss each of these using the SCCS example. Other examples of these approaches can be found on pages 145–147 in [11].

### 2.1 Covariate Stratification

Nonproportionality in Treatment can be addressed by fitting a separate baseline hazard function for each level of Treatment. The results for the SCCS data are given in Table 3. In this model, the effects of covariates are the same on each stratum so there is only one set of coefficients that apply to all strata. A disadvantage of this approach is that we cannot test the effect of treatment 1 versus treatment 2. For a variable that can be controlled this would make this approach unacceptable. It could, however, be used if a covariate such as race had a time varying coefficient.

**Table 3:** Parameter estimates for the proportional hazards model after stratification on Treatment

	coef	exp(coef)	se(coef)	z	P
RaceW	-0.87	0.42	0.30	-2.93	0.00
Stage2	-1.03	0.36	0.39	-2.65	0.01
Stage3	0.09	1.09	0.34	0.26	0.80

### 2.2 Partitioning of the Time Axis

Proportional hazards may not hold over the entire time axis but may hold approximately over shorter time periods. For the SCCS data we partition the time axis into two intervals: *t *< 30 and *t *≥ 30. The estimated coefficients for these two time periods appear in Tables 4 and 5. The parameters in the two time periods have been estimated separately so that each covariate can relate differently to survival during the two time periods.

**Table 4:** Parameter estimates for the proportional hazards model on the first time interval

	coef	exp(coef)	se(coef)	z	P
Treatment 2	-0.87	0.42	0.37	-2.39	0.02
RaceW	-0.61	0.54	0.34	-1.81	0.07
Stage2	-0.38	0.68	0.48	-0.79	0.43
Stage 3	0.26	1.30	0.39	0.67	0.50

**Table 5:** Parameter estimates for the proportional hazards model on the second time interval

	coef	exp(coef)	se(coef)	z	P
Treatment2	0.50	1.65	0.84	0.60	0.55
RaceW	-1.29	0.28	0.72	-1.79	0.07
Stage 2	-1.22	0.30	0.86	-1.42	0.16
Stage3	-0.80	0.45	0.87	-0.91	0.36

**Figure 5:**
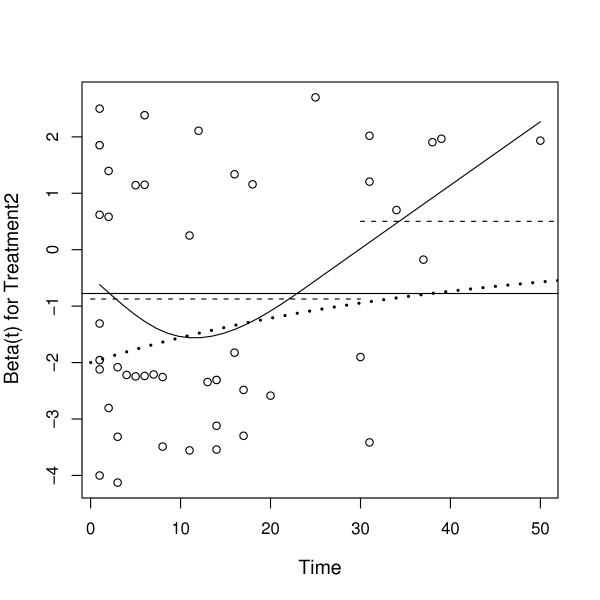
**Estimates for the dependence of *β*_Trt2 _on time**. The dependence of *β*_Trt2 _in the proportional hazards model (solid horizontal line), in the time-partitioned model (dashed line segments), and from a nonparametric estimate based on the Schoenfeld residuals (solid curve). The dotted curve is the actual dependence of *β*_Trt2 _on time (which is known in this case because the data have been simulated).

An advantage of partitioning the time axis is that we can model the effect of the Treatment variable. The relationship between this model and the proportional hazards model is illustrated in Figure 5 where the horizontal line indicates the coefficient *β*_Trt2_(*t*) is constant in time, the proportional hazards assumption, and the step function breaking at time *t *= 30 indicates *β*_Trt2_(*t*) is constant in time but only within each interval of the partition. The step function, like the curve showing the actual dependence on time, is increasing.

### 2.3 Modeling Time Dependence of *β*(*t*)

There are two basic approaches to modeling the dependence of *β*(*t*) on *t*. One is to specify a known functional form for this relationship. For example,

*β*_Trt2_(*t*) = *β*_0 _exp(-*ρt*)     (3)

so that the treatment effect diminishes over time. Another is to allow the data to select the functional form of the time dependence. This can be done by using splines. The basic idea is to replace each of the dashed line segments in Figure 5 with a curve such that the curves are connected at time *t *= 30. The time axis in Figure 5 is partitioned into just two intervals but by increasing the number of intervals a close approximation can be obtained.

We do not use this approach for the SCCS data. One reason is that software for this approach is not readily available. The SAS procedure phreg can be used when the known functional form is linear; however, this will not work for the function in equation (3). A more important reason is that allowing the data to choose the functional form increases the chance of over-fitting the data, and one must be cautious about interpreting the *p*-values and confidence intervals for the parameters.

### 2.4 Other Methods

The methods given above for dealing with nonproportional hazards all involve modification of the Cox model. Another approach is to simply use a different model. Therneau and Grambsch [11] suggest accelerated failure time or additive hazard models. In the Cox model, the covariates act multiplicatively on the baseline hazard function (1). The additive hazard model:

h(t;X)=h0(t)+∑j=1pβj(t)Xj(t)
 MathType@MTEF@5@5@+=feaafiart1ev1aaatCvAUfKttLearuWrP9MDH5MBPbIqV92AaeXatLxBI9gBaebbnrfifHhDYfgasaacH8akY=wiFfYdH8Gipec8Eeeu0xXdbba9frFj0=OqFfea0dXdd9vqai=hGuQ8kuc9pgc9s8qqaq=dirpe0xb9q8qiLsFr0=vr0=vr0dc8meaabaqaciaacaGaaeqabaqabeGadaaakeaacqWGObaAcqGGOaakcqWG0baDcqGG7aWocqWGybawcqGGPaqkcqGH9aqpcqWGObaAdaWgaaWcbaGaeGimaadabeaakiabcIcaOiabdsha0jabcMcaPiabgUcaRmaaqahabaacciGae8NSdi2aaSbaaSqaaiabdQgaQbqabaGccqGGOaakcqWG0baDcqGGPaqkcqWGybawdaWgaaWcbaGaemOAaOgabeaakiabcIcaOiabdsha0jabcMcaPaWcbaGaemOAaOMaeyypa0JaeGymaedabaGaemiCaahaniabggHiLdaaaa@4E32@

allows the covariates to modify the baseline hazard in an additive way; the effect of the covariate may also vary with time. The additive model measures the additional risk due to the effect of a covariate in *absolute *terms, whereas the Cox model measures it in *relative *terms.

Artificial Neural Networks (ANNs) are another class of models that have been used to model cancer data. These are sometimes referred to as "black box" models because of the complex, nonlinear relationships used in the fitting procedure. In the Cox model, the form of the relationship between the covariates and survival is specified; only the parameters of the model are estimated. In contrast to this, with ANNs one tries to estimate both the functional form of the relationships between variables and the parameters that describe those relationships. This limits the ability of these models to identify causal relationships, because it is usually difficult to relate the model parameters to the biological context that generated the data (Ahmed [12]).

Tree Structured Survival Analysis (TSSA) provides another alternative for the study of risk factors (Segal [13]). It is an extension of the Classification And Regression Tree (CART) algorithm developed by Breiman *et al *[14]. TSSA is an exploratory, nonparametric method that requires no assumptions about the relationship between survival and the potential risk factors (covariates). The primary output of a TSSA algorithm is a "tree structure", whose branches and nodes identify the successive splits of the cases into risk groups with similar survival profiles. TSSA evaluates the relationship between risk factors and survival through recursive partitioning of patients according to their risk factors, with a comparison of survival patterns among the subgroups of patients resulting from each partition. The method not only identifies a set of significant risk factors, but also provides a simple procedure to identify subgroups of participants with an estimate of their associated risk.

## 3 Application of Modified Cox Models in Colon Cancer Research

Moreau *et al *[15] proposed a crude survival model that accounts for the nonproportionality of hazards by modeling the hazard ratio as a step function of the follow-up time (divided into predefined intervals). The application of the piecewise model requires determining both the number and the boundaries of these intervals. Clinicians have conveniently suggested six intervals with the following upper boundaries being 1 month, 6 months, 12 months, 24 months, 60 months, and 120 months (Bolard *et al *[16]), and employed the 2L program of BMDP Statistical Software analysis (Dixon *et al *[17]).

Esteve *et al *[18] fit the baseline hazard function and its dependence on time by partitioning the time axis. However, the coefficients of the covariates were not allowed to depend on time. In contrast, Moreau *et al *[15] allow the coefficient on age to depend on time and finds the effect of age continues to decline between 24 and 120 months. Moreau *et al *[15] also allow cancer stage to depend on time. These considerable differences between models that allow covariates to depend on time and those that do not demonstrate the ability of models with time varying coefficients to provide new insights about these changes.

Esteve *et al *[18] investigate the time-dependent logarithm of the hazard ratio for each covariate by modeling the data using B-splines (de Boor [19] and Stone and Koo [20]). Using this B-spline model, they find a significantly higher risk of post surgical mortality than the Cox model during the first six months, and by 12 months the reverse happens. Moreover, the Cox model overestimates the impact of age on colon cancer-specific mortality after the first 6 months of follow-up, whereas the impact of age is near zero during this time period when the B-spline model is used. Clinically, the risk of cancer related death for elderly patients is higher only during the initial few months corresponding to post-surgical period. The estimated effect of later periods of diagnosis shows that there may be some benefits of new treatments in reducing post-surgical mortality, but they do not appear to affect the long-term survival (Giorgi *et al *[21]).

## 4 Software

Software routines for fitting Cox models are available in all of the popular statistical software packages. Here we provide only a brief summary for some of those that are more widely used. It is worth noting that many of these programs have active and well-organized user groups whose members are continually writing add-on functions and macros, usually available at no cost.

### GLIM

GLIM [22], the Generalised Linear Interactive Modeling package, is a flexible, interactive statistical analysis program developed by the GLIM working party of the Royal Statistical Society. It has a suite of macros that perform survival analysis. These include COXMODEL (fits Cox proportional hazards models), PHAZ (plots log hazard against log survival time), LIFETABS (produces a life table analysis from censored survival times), and STEPS (plots the estimated survival curve using the Kaplan-Meier approach). Other related macros are INIB, LOGRANK2, NORM, LNORM, WEIBMIX, WEIBULL, and RESPLOTS.

### R

R [23] is a language and environment for statistical computing and graphics. Unlike the other packages mentioned here, R can be downloaded at no cost from the R website. It is similar to the S language and environment which was developed at Bell Laboratories (formerly AT&T, now Lucent Technologies). Much code written for S runs unaltered under R. Analysis is accomplished via calls to functions; the arguments to these functions include datasets and model specifications, and the function returns appropriate statistics and graphical output. An R add-on package called "survival" (again, available at no cost) contains over 100 files consisting of functions and datasets for survival analysis.

### SAS

SAS [24] software has six procedures that perform survival analysis computations. LIFETEST produces life tables and graphs of survival curves. LIFEREG estimates regression models with censored data, but does not allow for time-dependent covariates. PHREG fits Cox models, handling both discrete-time and continuous-time data, as well as time-dependent covariates. Three other procedures (LOGISTIC, PROBIT, and GENMOD), while not designed specifically for survival analysis, are effective for estimating survival models in certain settings.

### S-PLUS

Marketed by Insightful Corporation, S-PLUS [25] contains a complete array of survival analysis tools, including frailty models, smoothing splines, penalized survival models, parametric survival regression, Kaplan-Meier curves, and Cox proportional hazards models.

### SPSS

SPSS [26] is a popular menu-driven general statistical analysis package. It contains menu-driven routines for constructing Life Tables, plotting Kaplan-Meier survival curves, and fitting Cox models with time-varying covariates.

### Other Programs

Though less widely used than those we have mentioned above, other programs that have Cox model fitting capabilities include BMDP [27], NCSS [28], Minitab [29], Systat [30], Stata [31], and Statistica [32].

## Conclusion

The Cox model, also known as the proportional hazards model, often provides a very good approximation to the survival function and its dependence on covariates. For those situations where the proportional hazards model is inadequate we have described three simple modifications that address nonproportional hazards. One solution is to stratify covariates. A disadvantage of this approach is that it does allow modeling the effect of the covariates used in the stratification. Another solution is to partition the time axis into intervals and require proportional hazards only within intervals. A disadvantage here is determining the number intervals that are necessary. The final approach is to model the time dependence of the coefficients. Interpretation should be tempered by the fact that these models tend to over-fit the data.
